# 

**Published:** 1915-12-04

**Authors:** 


### Buildings Contemplated.

Arbroath.—The Arbroath local authorities have applied to the Treasury for sanction to borrow £2,000 to complete and furnish the smallpox hospital.

Bracebridge.—At a meeting of the Holland County Council at Spalding it was decided to proceed with the extensions to Bracebridge Asylum, Lincoln, and to meet the cost out of the current revenue.

Brandon.—The Brandon and Byshottles Urban District Council have instructed the surveyor to prepare amended plans for an isolation hospital in Sleetburn Road.

Chelsea.—An appeal is being made for funds for the completion of the new Chelsea Hospital for Women and Nurses' Home, for which a sum of £35,000 is still required.

Lambeth.—The Guardians have decided to convert part of a workhouse into an infirmary for 300 patients. The accommodation is required for patients to be transferred from the south infirmary, which has been acquired for a war hospital.

Leeds.—The enlargement of the 2nd Northern General Hospital at Beckett's Park by the erection of additional buildings on the college playing fields is under consideration.

Oakdale.—The new cottage hospital at Oakdale, near Blackwood, has been formally opened. The architect was Mr. Webb, of Blackwood, and the builders were Messrs. Lewis Bros., of Bargoed. The cost was £4,500.

Truro.—An agreement has been completed between the Guardians and the Red Cross Society for the taking over of the workhouse as a naval hospital.

Wells.—The Somerset County Council have requested the Wells Rural District Council to acquire a site and to prepare plans for an isolation hospital.

### PASSING EVENTS AND TOPICS.

### Southampton Hospital Sunday Fund.

The total amount collected this year for the Southampton Hospital Sunday Fund is stated to be £1,484, the largest sum raised in one year since the Fund was inaugurated forty-four years ago.

### Royal Hospital for Incurables.

At the annual meeting of the Royal Hospital for Incurables, Putney Heath, held on November 26, it was stated that the income had declined from £62,909 in 1914 to £31,186 this year, and that unless there was an early improvement in the financial receipts curtailment in the future number of beneficiaries might be necessary.

### A Twilight Sleep Hospital.

According to the New York *Medical Record*, the Executive Committee of the Twilight Sleep Association has decided to establish a hospital where treatment by this method can be given. It is the belief of the Association that interest in the "twilight sleep" propaganda will be increased by the creation of a centre for treatment, and for the instruction of physicians.

### Tuberculosis and Chemical Fumes.

A New York physician has addressed a letter to the health authorities of that city, in which he expresses the belief that the fumes from certain chemical factories are active factors in the spread of tuberculosis. Not only do the fumes aggravate the condition of those suffering from respiratory diseases, but they compel people living in the neighbourhood of the factories to keep their windows closed.

### Royal Inspection of Motor Ambulance Cars.

On November 25 H.M. the Queen, on behalf of the King, inspected in the garden of Buckingham Palace a convoy of motor ambulance cars which have been presented to the British Red Cross Society in England by the Calcutta "Statesman" War Fund. On the same day Her Majesty visited the sick and wounded officers who have returned from the Front at the Convalescent Hospital for Officers, 83 Portland Place.

### Eggs for the Wounded.

The proposal that kindly disposed members of the public should contribute new-laid eggs for the consumption of wounded soldiers has met with a very gratifying success. The requirements of the various hospitals at the moment at the base in France and throughout this country are approximately 700,000 eggs per week.

### Sterilisation of Water for the Troops.

An article in a Military Supplement of the *Muenchener Medizinische Wochenschrift* on this subject pronounces most of the usual methods of sterilising water —such as filters, boiling, pasteurising, and the use of ultra-violet rays—as defective for wholesale use. For campaign purposes it is contended that it is best to rely upon chemical germicides, and that the best of these is probably chlorinated lime (thirteen parts in 100,000), which in ten minutes will destroy all life in the water; the chlorine is removed from the water by sulphates or peroxides. For use in the field two sets of tubes are provided, the one containing chlorinated lime and the other an oxidising substance.

### Brompton Hospital and Tuberculous Soldiers.

At an interim court of governors of the Hospital for Consumption and Diseases of the Chest, Brompton, held on November 26, the Duke of Wellington, who presided, drew attention to the fact that the facilities afforded to the authorities for the admission of sailors and soldiers discharged from the Services suffering from consumption continued to be made full use of with great advantage to the patients concerned. Many of these it had been possible to transfer to the Frimley Sanatorium in order to undergo special treatment. The number of discharged sailors and soldiers now under treatment is nearly a hundred.

### The Miners as Methodical Givers.

The miners of Senghenydd have sent £40 to cover their contributions to the King Edward VII.'s Hospital, Cardiff, for the past two months. For the future they will send £20 monthly. The Secretary (Mr. Rea), with Mr. Rowland Evans, recently addressed a meeting at Senghenydd, and the £40 was the result of eight weeks' collections on the penny per man per week system. As the annual contribution from Senghenydd had previously amounted to from £40 to £50, it would mean that their subscription would now be increased five-fold, or by £200 per annum. It is considered that if this plan were adopted throughout the coalfield the hospital would be freed for all time from financial embarrassment.

### Munition Workers and Hospitals.

At the annual meeting of the North Lonsdale Hospital, Barrow, where some 718 in-patients were treated during the year, the receipts amounted to nearly £5,000, £1,400 of which was devoted to the building account. The hospital authorities have had a difficulty in securing a resident medical officer, and the work has been undertaken by Dr. J. T. Williams, a member of the consulting staff, who had retired from the hospital after forty-one years' service. Much gratitude was expressed to Dr. Williams, and to the matron, Miss Summers, who have managed the affairs of the hospital excellently well despite the difficult circumstances of the year. It is remarkable and encouraging that this hospital obtained an additional sum of £725 by contributions from the munition war workers who had taken up their residence in Barrow.

### Infirmary Treatment of Insured Consumptives.

The attention of the Sanatorium Benefit Sub-Committee of the London Insurance Committee has been drawn to the difficulties experienced by insured persons desirous of entering infirmaries on the expiration of their period of treatment in a sanatorium or hospital owing to the fact that the Board of Guardians responsible for finding accommodation for them cannot easily be ascertained. The Metropolitan Asylums Board are proposing to approach the Local Government Board with a view to arrangements being made whereby a destitute and infirm insured person may be in a position to obtain immediate admission to an infirmary on leaving a general hospital or sanatorium, and at a meeting of the London Insurance Committee on November 25 a report was submitted by the Sanatorium Benefit Sub-Committee, in which it was suggested that the Sub-Committee should make similar arrangements.

### Personalia.

Mr. G. Ruddle, secretary of the Salford Royal Hospital, has been elected to succeed Mr. W. G. Carnt (who held the position since the inception of the Association eight years ago) as president of the Northern Association of Hospital Secretaries

Dr. T. E. Richards, medical officer of health to the Ystradgynlais Rural District Council, has resigned that position.

Dr. Oliver, resident medical officer; Dr. Buckley, accident-room surgeon; and Dr. Hughes, resident surgical officer, have resigned their posts at the Manchester Royal Infirmary to go on active service.

Mr. James Orr, L.R.C.P., L.R.C.S. (Edin.), medical officer of health for Barrow-in-Furness, has withdrawn his request for leave to join the R.A.M.C., owing to the strongly expressed opinions of members of the Corporation and the medical department of the Local Government Board.

Dr. E. C. Lambert, assistant medical officer for the Swaffield Road Institution, has notified the Wandsworth Board of Guardians that he has accepted a temporary commission in the R.A.M.C., and has arranged, subject to their consent, for Dr. Thomas, of Garratt Lane, Wandsworth, to carry on his work.

Mr. J. P. Jones, late clinical assistant at the Lambeth Infirmary, has been engaged as *locum tenens* by the Lambeth Board of Guardians. Dr. Baly, the medical superintendent, informed the Board that Dr. Jones rendered most useful service at the time when the patients were being transferred from the Southwark Infirmary when the latter was taken over by the military authorities.

### Bury Infirmary.

The following have been appointed as honorary assistant surgeons on the staff of the Bury Infirmary: Dr. Alexander Stewart, of Mayfield, Bury, graduate of the Glasgow University; Dr. Stewart acted both as house surgeon and house physician in the Royal Victoria Infirmary, Glasgow, and was for some time senior resident surgeon to the Royal Halifax Infirmary. Dr. C. G. Lees, of The Rowans, Bury, graduate of Victoria University, Manchester; Dr. Lees acted for some months as house surgeon to the North Staffordshire Infirmary, Stoke-on-Trent.

### Gifts and Bequests.

Miss Ann Heap, of Tavistock Square, London, bequeathed £500 to the Royal Hospital for Incurables, Putney, and £100 each to the Cottage Hospital, Lutterworth, the Homœopathic Hospital, Leicester, and the Homœopathic Hospital, Great Ormond Street.

Mr. Donald Fisher, of Edinburgh, bequeathed £200 to the Edinburgh Royal Infirmary, and £100 each to the Royal Blind Asylum and School, Edinburgh, the Royal Edinburgh Hospital for Incurables, and the Scottish National Institution for the Education of Imbecile Children.

Mr. Edwin Carr, of Leicester, proprietor of Carr's fever powders, bequeathed £5,000 to the Royal Infirmary, Leicester; £2,000 to the Children's Hospital; and £100 each to the Faire Hospital and the Maternity Hospital. One half of the residue after various other bequests is to go to the Leicester Infirmary and the other half to charitable objects to be selected by the executors.

Mr. Joshua Preston, J.P., head of the firm of wholesale clothiers, Messrs. Joshua Preston and Son, Ltd., Stockport, has given £500 for the endowment of a cot in the Stockport Infirmary as a memorial to Nurse Cavell. The cot will bear the inscription: "This cot was endowed by Joshua Preston, Esq., J.P., of Stockport, in memory of the devotion and heroism of Nurse Edith Cavell, October 1915."

### Medical Appointments.

St. Thomas's Hospital.—The following appointments, dated January 1916, have been made: *Resident Assistant Physician*, Mr. Geoffrey Hoffmann, B.A., M.B., B.C. Cantab., M.R.C.P. Lond.; *Medical Registrar*, Mr. J. L. Birley, M.A., M.B., B.Ch. Oxon., M.R.C.P. Lond.; *Surgical Registrar*, Mr. P. H. Mitchiner, M.B., M.S. Lond., F.R.C.S. Eng.; *Obstetric Registrar*, Mr. J. M. Wyatt, M.B., B.S. Lond., F.R.C.S. Eng.; *Ophthalmic Registrar*, Mr. P. G. Doyne, B.A., M.B., B.Ch. Oxon., F.R.C.S. Eng.

Manchester Royal Infirmary.—The following appointments have been made: *Assistant Surgical Officers*, Messrs. E. E. Hughes and John Gow; *Accident-room House Surgeon*, Mr. A. H. Southam; *Medical Officer* (central branch), Mr. J. H. Chorlton; *Resident Medical Officer*, Mr. Charles E. Thornton; *Surgical Officer* (out-patient department), Mr. H. Buck.

Rates of Subscription: Twelve months, 6/6; Six months, 4/-; Three months, 2/-, post free to any address in the United Kingdom. Abroad, Twelve months, 8/8; Six months, 5/-; Three months, 2/6, post free.—Address The Subscription Dept., "The Hospital," The Hospital Building, 28/29 Southampton Street, Strand, London, W.C.

